# 

**Published:** 1885-08

**Authors:** 


### BOOKS RECEIVED.

---

*Compendium der Ohrenheilkunde.* Von Dr. A. Sarron. Leipzig Ambr. Abel.

*The Nature of Mind and Human Automatism*, by Morton Prince, M. D.

*Micro-Chemistry of Poisons, including their Physiological, Pathological, and Legal Relations*, with an Appendix, by Theodore G. Wormley, M. D., Ph. D., LL. D. Second edition. Philadelphia : J. B. Lippincott & Co.; Chicago : W. T. Keener.

*A General Index to the First Twenty Volumes of St. Bartholomew's Hospital Reports from 1865 to 1884.* London : Smith, Elder & Co.

*Urinary and Renal Derangements and Calculous Disorders*, by Leonel S. Beale, M. D. Philadelphia : Blakiston Son & Co. ; Chicago : W. T. Keener.

*Hay-Fever*, by Charles E. Sajons, M. D. Philadelphia : F. A. Davis, Attorney.

*A Treatise on Asiatic Cholera.* Edited and Prepared by Edmund Charles Wendt, M. D. New York : William Wood & Co.; Chicago: W. T. Keener.

*A Manual for Hospital Nurses and others Engaged in Attending on the Sick*, by Edward J. Domville. Philadelphia: P. Blakiston, Son & Co. ; Chicago: W. T. Keener.

*A Treatise on Practical Chemistry*, by Frank Clowes, D. Sc. Philadelphia: Lea Bros. & Co.; Chicago: Jansen, McClurg & Co.

*A Treatise on the Science and Practice of Midwifery*, by W. S. Playfair, M. D., F. R. C. P. Fourth American edition. Philadelphia: Lea Bros. & Co. ; Chicago: Jansen, McClurg & Co.

*The Rational Treatment of Rupture*, by A. H. Parker, M. D., Chicago.

*Report on the Mortality and Vital Statistics of the United States as returned at the Tenth Census*, by John S. Billings, Surgeon U. S. Army.

*A Practical Treatise on Urinary and Renal Diseases including Urinary Deposits*, by William Roberts, M. D., F. R. S. Philadelphia: Lea Bros. & Co. ; Chicago: Jansen, McClurg & Co.

*Second Report of the State Board of Health of the State of Tennessee.*

### PAMPHLETS RECEIVED.

Proceedings of the Nineteenth Annual Meeting of the Alumni Association of the Chicago Medical College.

Prospectus of the Oakwood Retreat, Lake Geneva, Wisconsin.

Sanitary Suggestions or How to Disinfect our Homes.

A Case of Extensive Recurrent Sarcomatous Disease. By John S. Miller, M. D.

Lectures on Phthisis Pulmonalis. By Ernest L. Shurley, M. D.

Medical Legislation. By Henry O. Marcy, A. M., M. D.

Report on Practical Medicine. By Ira E. Oatman, M. D.

Report of the Board of Trustees of the Michigan Asylum.

Electricity as a Remedial Agent. By George C. Pitzer, M. D.

A Word in Regard to the Pennsylvania State Board of Health Bill. By R. Harvey Reed, M. D.

Vitiligo. By R. Harvey Reed, M. D.

The Ohio State Sanitary Association. Second Annual Meeting.

Sanitary Schedule for State Sanitary Survey. Illinois State Board of Health.

Some Interesting Reflex Neuroses. By John J. Caldwell, M. D.

Twenty-fourth Annual Report of the Cincinnati Hospital.

Asiatic Cholera. By Oscar C. De Wolf, A. M., M. D.

Forty-second Annual Report of the Managers of the State Lunatic Asylum at Utica, New York.

Thirty-sixth Annual Report of the Trustees and Superintendent of the Indiana Hospital for the Insane.

Restraint or Non-restraint in the Treatment of the Insane. By Dr. W. B. Fletcher.

The Physiological Effects and Therapeutical Uses of Hydrastis. By Roberts Bartholow, M. D., LL. D.

Special Report on the Hungerford Outbreak. Arthur S. Hardy, Secretary.

Vital Statistics in Tennessee. A Report by J. B. Plunket, M. D.

Specialties and their Relation to the Medical Profession. By L. Duncan Bulkley, A.M., M. D.

"An Enemy Came and Sowed Tares." By Joseph Eastman, M. D.

Endometritis Fungosa. By James B. Hunter, M. D.

Constitutional Treatment of Caries and Necrosis. By Hal. C. Wyman, M. D.

History of the Clamp-Suture of the late Dr .J. Marion Sims, and Why it was Abandoned by the Profession. By Nathan Bozeman, M. D.

The Over-crowding of the Profession. By Dr. E. J. Doering, M. D.

Suerseus Obturators. By Dr. L. Th. Weber.

Medical Society of the State of Tennessee. Transactions 1885.

Pneumonia in Young Children. By L. Emmett Holt, A. M., M. D.

Does Quinine Abort Pneumonia? By L. Emmet Holt, A. M., M. D.

Hydatid Tumors of the Brain. By R. Harvey Reed, M. D.

Cholera, its Nature, Symptoms, History, Cause and Prevention. By J. B. McConnell, M. D.

SUBSCRIPTION PRICE, $3.00 PER YEAR.

Whole Number, 308. AUGUST, 1885. Vol. LI., Number 2.

INDEX MEDICUS

### THE CHICAGO MEDICAL JOURNAL AND EXAMINER

LIBRARY INDEXED S.G.O.

EDITORS:

S. J. JONES, M.D., LL.D.

W. W. JAGGARD, A.M., M.D. HAROLD N. MOYER, M.D.

COLLABORATORS.

CHICAGO:

PROFESSOR J. A. ALLEN, M. D., LL. D.
PROFESSOR E. ANDREWS, M. D., LL. D.
PROFESSOR W. H. BYFORD, A. M., M. D.
PROFESSOR O. C. DEWOLF, A. M., M. D.
PROFESSOR E. C. DUDLEY, A. B., M. D.
PROFESSOR J. H. ETHERIDGE, A. M., M. D.
PROFESSOR C. FENGER, M. D.
PROFESSOR J. N. HYDE, A. M., M. D.
PROFESSOR E. FLETCHER INGALS, M.D.
PROFESSOR H. A. JOHNSON, M. D., LL. D.
CHARLES GILMAN SMITH, A. M., M.D
J. F. TODD, M. D.

MILWAUKEE, WISCONSIN:
PROFESSOR N. SENN, M. D.

WASHINGTON, D. C:
S. O. RICHEY, M. D.

UNITED STATES NAVY:
MEDICAL DIRECTOR J. M. BROWNE, M. D.

CHICAGO:

